# Optimizing Conditions for *Bacillus subtilis* Ectopic Gene Expression and Delivery via Seed Treatment

**DOI:** 10.3390/plants14203184

**Published:** 2025-10-16

**Authors:** Abeer Alnasrawi, Jiamei Li, Payal Sanadhya, J. Alejandro Rojas, Fiona L. Goggin

**Affiliations:** 1 Department of Entomology and Plant Pathology, University of Arkansas, Fayetteville, AR 72701, USA; abeeralnasrawi@gmail.com (A.A.); jxl080@uark.edu (J.L.); sanadhya@uark.edu (P.S.); 2 Cell and Molecular Biology Graduate Program, University of Arkansas, Fayetteville, AR 72701, USA; 3 Department of Plant, Soil and Microbial Sciences, Michigan State University, East Lansing, MI 48824, USA; rojasfle@msu.edu

**Keywords:** seed coating, plant elicitor peptide, *Bacillus subtilis*, biocontrol, plant growth promoting bacteria, rhizobacteria

## Abstract

*Bacillus subtilis* is applied as a biofertilizer, biocontrol agent, and probiotic in agriculture, and is also used for industrial synthesis of proteins and peptides. These applications can be combined by using *B. subtilis* to synthesize plant health-promoting peptides in culture or to deliver them to roots via seed treatments. To facilitate the use of *B. subtilis* as a cell factory, we tested different media, temperatures, and growth phases to optimize ectopic expression of a Plant Elicitor Peptide from soybean (GmPEP3) that enhances seedling growth. Our results indicate that temperature, culture media, and growth phase have interactive effects, and that 30 °C and 2x YT media can enhance ectopic expression per cell compared to 37 °C or LB media in log phase bacteria. We also identified tradeoffs between cell growth and ectopic expression levels per cell, with the log phase favoring high expression per cell but the stationary phase yielding higher cell numbers and consequently higher expression levels per unit of growth media. In addition, to facilitate *B. subtilis* seed treatments, we compared retention of spores versus vegetative cells with and without carboxymethylcellulose (CMC) to improve the viability of *B. subtilis* seed treatments. Our results indicated that retention of viable bacteria on *B. subtilis*-treated seeds could be increased by ~40% by using the adhesive polymer CMC, and shelf life could be extended from 24 h to at least 3 months by using endospores rather than vegetative cells. For *B. subtilis* expressing GmPEP3, endospores also had comparable plant-growth-promoting activity as vegetative cells. This establishes the bioactivity of spores and illustrates the potential benefits of using *B. subtilis* to deliver heterologous peptides. These results provide valuable insights for deploying *B. subtilis* for crop health.

## 1. Introduction

*Bacillus subtilis* or the grass bacillus is an aerobic, Gram-positive rhizobacterium that forms endospores, a resting stage that facilitates survival of nutrient starvation or challenging environmental conditions such as extreme temperatures or low water availability [1]. In part due to its ability to produce these long-lived spores, *B. subtilis* is found in a diverse array of environments, from soil, air, and oceans to the roots of plants and the guts of humans and bees [1,2]. Naturally occurring *B. subtilis* isolates are utilized as biostimulants and biocontrol agents for crops, as well as probiotics for livestock and humans [3,4]. Another largely separate body of research has developed *B. subtilis* as a cell factory for commercial synthesis of peptides and proteins, including pharmaceutical antigens, antibodies, antimicrobial peptides, and enzymes used in food processing, detergents, and textile production [5]. Bringing these perspectives together has great potential to facilitate the use of *B. subtilis* for synthesis and delivery of bioactive peptides that enhance plant growth and promote crop protection.

Many known peptides have potential applications in crop production as plant growth regulators, immune activators, and antimicrobials [6]; however, in order to deploy these peptides in agriculture, we need affordable synthesis methods and delivery systems that can overcome the sensitivity of peptides to degradation by heat and UV light [6]. Several characteristics of *B. subtilis* make it particularly suitable for either commercial peptide synthesis in bioreactors or on-plant synthesis and delivery of heterologous peptides and proteins. This species is highly amenable to genetic modification, with effective protocols available not only for classical selection marker-based transformation, but also for genetic engineering with counter-selectable markers [7], site-specific recombination systems [8], and the CRISPR-Cas9 system [9]. *B. subtilis* also has a rapid growth rate, enabling peptide or protein synthesis in only one fourth of the incubation time needed for expression in *Saccharomyces cerevisiae*, another common system for recombinant expression [5]. In addition, compared to other bacterial expression systems such as *Escherichia coli*, *B. subtilis* can much more readily be engineered to secrete recombinant proteins and peptides [5,10]. This greatly improves the efficiency of industrial protein production because it eliminates the need for cell lysis and removal of undesirable cell contents. In addition, the secretory capabilities of *B. subtilis* create opportunities to use the bacteria itself to deliver heterologous peptides to plant roots.

Like other Plant Growth-Promoting Rhizobacteria, *B. subtilis* forms biofilms on root surfaces from a wide variety of plant species and also colonizes the surrounding rhizosphere [11,12]. This positions it well to deliver peptides and other compounds to crops. In fact, naturally occurring *B. subtilis* strains use multiple secretory pathways to deliver a wide variety of endogenous peptides, enzymes, and other biomolecules to the rhizosphere [3,13]. *B. subtilis* also establishes a long-term relationship with roots, allowing it to deliver peptides over the course of the entire growing season and overcome the typically short environmental lifespan of these molecules. Thus, *B. subtilis* represents a promising delivery system for recombinant peptides and proteins.

As a test case for this approach, we previously utilized *B. subtilis* to express a signaling peptide from soybean (Plant Elicitor Peptide 3, or GmPEP3) and deliver it to soybean roots through seed treatments and root drenches [14]. PEPs are signaling molecules found throughout higher plants that modulate plant growth and defense, and that can be applied to plants to prime immune responses against pests and pathogens [15]. Compared to plants treated with a *B. subtilis* empty vector control that lacked GmPEP3, soybean plants treated with vegetative cells of the engineered bacteria had significantly more rapid seedling growth and decreased infection by the soybean cyst nematode *Heterodera glycines* [14]. These results and similar findings in potato [16] demonstrate that *B. subtilis* can enhance crop growth and immunity through delivery of recombinant peptides. These prior results provide a foundation for future work to optimize the conditions for recombinant peptide expression in *B. subtilis* and the methods for *B. subtilis* seed treatments.

To optimize conditions for ectopic expression in bacterial systems, three important variables to consider are the growth phase in which bacteria are harvested and the choice of culture media and incubation temperature. Heterologous enzyme production is reported to be higher in the stationary phase than in the log phase in *B. subtilis* [17] and to be enhanced by Yeast Extract Tryptone media (2x YT) compared to Standard Media or Luria–Bertani (LB) media in *B. subtilis* and *E. coli* [18,19]. Studies in *E. coli* also suggest that protein production is higher at 30 °C than at 37 °C even though cell growth is faster at 37 °C [20,21]. While these separate studies on different bacterial growth conditions provide a useful starting point for optimizing ectopic expression in *B. subtilis*, further work is needed to examine how growth phase, media, and temperature interact to influence expression levels in this species.

In order to utilize *B. subtilis* for direct delivery of recombinant peptides to roots, we also need effective, economical strategies to inoculate crops with the bacteria. Seed treatments are a particularly promising means of delivering microbial bioprotectants like *B. subtilis* because they require less inoculum and labor than root or soil drenches, are more readily scalable for large-acreage crops, and can colonize the plant as soon as seeds germinate [22]. Furthermore, because of the widespread commercial adoption of seed coatings and seed-applications of pesticides [23], microbial seed treatments can more readily be pyramided with existing agronomic practice than root drenches. However, a major limitation in the use of biological seed treatments is the challenge of capturing and retaining high levels of viable bacteria on the seed until it is planted [24,25]. Formulations of *B. subtilis* probiotics for human consumption utilize endospores rather than vegetative cells because of their stability in storage and in extreme environments [26], and although relatively few agricultural studies directly compare these life stages, the use of bacterial endospores rather than vegetative cells for seed treatment has been proposed as a means of increasing bacterial survival of the spray-drying application process [27]. In addition, carboxymethylcellulose (CMC) is an inexpensive, nontoxic, and biodegradable polymer that can be used as a sticking agent to help microbial agents adhere to seeds, e.g., [28,29]. Therefore, the combined use of endospores and a polymer adhesive such as CMC could potentially enhance the retention of viable bacteria (i.e., shelf life) on *B. subtilis*-treated seeds.

The goals of this study were to help optimize the conditions for recombinant gene expression in *B. subtilis* as well as the methods for *B. subtilis* seed treatments. To optimize conditions for recombinant expression, we assessed how bacterial growth phase (stationary vs. log phase), media (2x YT vs. the more widely used LB media), and temperature (30 °C vs. 37 °C) interacted to influence viable cell numbers and transcript abundance of a heterologous gene (*GmPEP3*) in *B. subtilis* RIK1285. Our results indicated that harvesting cultures in the stationary phase yielded higher cell numbers per ml of culture than the log phase, whereas the log phase yielded significantly more heterologous transcripts per cell, particularly when grown at 30 °C and/or on 2x YT. The highest transcript abundance per unit of culture media was observed in the stationary phase grown on 2x YT at 30 °C. To facilitate the use of *B. subtilis* seed treatments, we also tested whether the shelf life of seed treatments could be improved by using *B. subtilis* endospores rather than vegetative cells, and/or by adding CMC. In addition, to confirm that endospores would have similar bioactivity as vegetative cells, we tested the effects of seed treatments with *GmPEP3*-expressing spores versus vegetative cells on the growth of soybean seedlings. Our results established that adding CMC and using endospores rather than vegetative cells for seed treatments both significantly enhanced the number of viable bacteria on seeds. Furthermore, endospores had comparable growth-promoting activity as viable vegetative cells. These results will facilitate the development of *B. subtilis*-based peptide expression and delivery systems for plant growth promotion and crop protection.

## 2. Results

### 2.1. Influence of Growth Phase and Growth Conditions on the Number of Viable B. subtilis Cells Ectopic Transcripts per Cell

**Figure 1:**
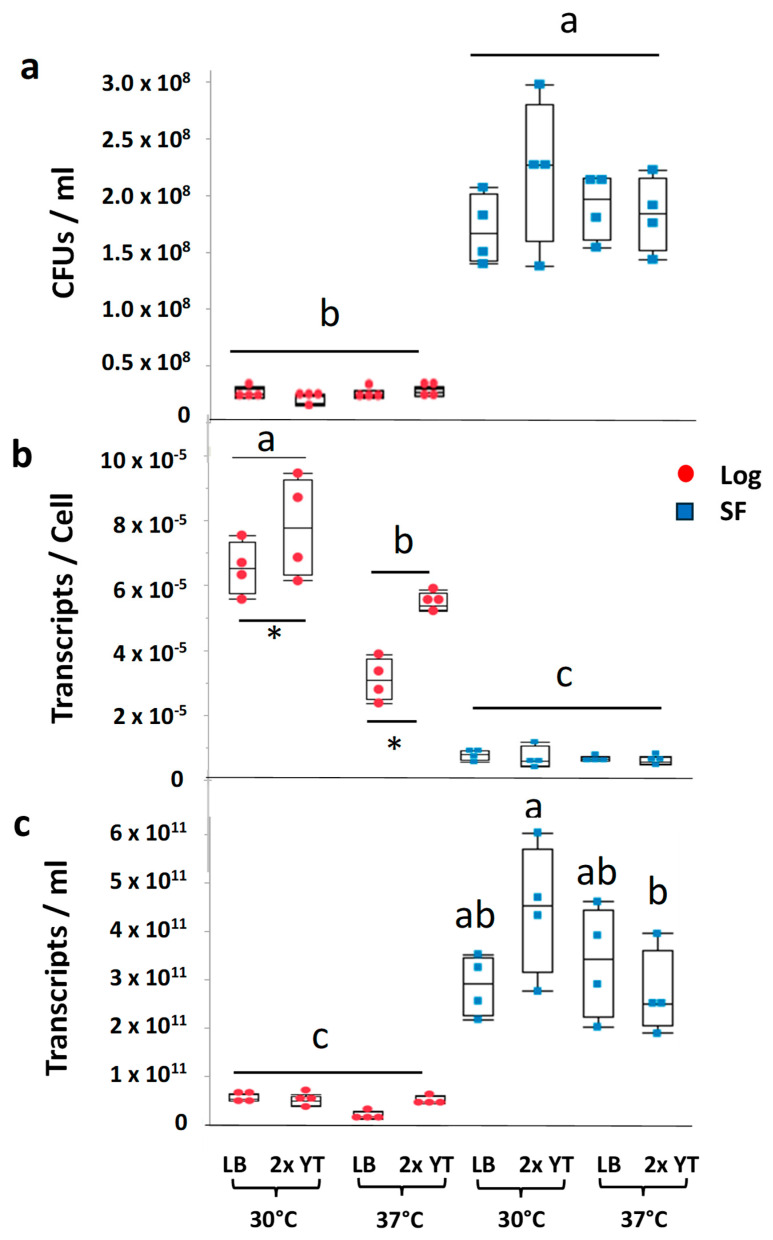
Effects of growth conditions on ectopic expression of GmPEP3 in *B. subtilis*. The number of colony-forming units (CFUs) for *B. subtilis* expressing *GmPEP3* at 30 °C and 37 °C on Luria (LB) and Yeast Extract Tryptone media (2x YT) in the log phase (LF) and stationary phase (SF) (**a**). Transcript abundance of *GmPEP3* per cell (**b**) and the abundance of *GmPEP3* transcripts per ml (**c**). The data represent the average of two subsamples for 4 biological replicates. For CFUs (**a**), the only statistically significant difference among treatment groups was a significant main effect of growth phase (Mixed model: *p* < 0.0001). For transcripts/cell (**b**), there was a significant interaction between growth phase and temperature, and letters report the results of Tukey’s Honestly Significant Difference (HSD) mean separation test (α = 0.05). In addition, there was a significant interaction between media and growth phase (*p* = 0.0006), with 2x YT yielding higher transcript abundance per cell than LB in the log phase only (represented by asterisks). For transcripts/mL of media (**c**), there was a significant interaction among all three variables, and letters report the results of Tukey’s Honestly Significant Difference (HSD) mean separation test (α = 0.05).

The first goal of this study was to examine how the bacterial growth phase (log phase or stationary phase), temperature (37 °C or 30 °C), and culture media (Luria broth [LB] or 2x Yeast Extract Tryptone medium [2x YT]) interact to influence the number of viable cells and the transcript abundance of a heterologous gene (*GmPEP3* from soybean) driven by the Subtilisin E (*aprE*) promoter commonly used in *B. subtilis* for ectopic expression [30]. The *B. subtilis* strain RIK1285 (Takara Bio Inc., San Jose, CA, USA) was utilized for this study because it is a well-established food-grade recombinant expression system [31]. To identify the time points at which to collect the log and stationary phases, we created growth curves for each possible combination of media and temperature. At 37 °C on either media, *B. subtilis* reached the midpoint of log phase (OD600 ~0.3) at 2 h and the stationary phase (OD600 ~0.7) at 8 h, whereas at 30 °C on either media, 4 h and 24 h were appropriate time points to collect the log and stationary phases (Figure S1). When the number of viable *B. subtilis* cells was then compared for all possible combinations of our three experimental variables by plating serial dilutions of each culture, we found that colony-forming units (CFUs) per ml of culture were significantly higher in the stationary phase than in the log phase (*p* < 0.001), and were not significantly affected by temperature, growth media, or the two- and three-way interactions among these factors (all *p* > 0.10) (Figure 1a). In contrast, when *GmPEP3* transcript abundance was measured using absolute real-time RT-PCR and the number of *GmPEP3* transcripts per viable cell was calculated, expression per cell was higher in the log phase than in the stationary phase (*p* < 0.001), and there were also significant interactions between growth phase and temperature (*p* < 0.001) and growth phase and media (*p* = 0.0006). In log phase cells, transcript abundance per cell was significantly enhanced by YT media compared with LB, and also enhanced by lower temperature (30 °C) compared with higher temperature (37 °C). In contrast, in the stationary phase, the abundance of *GmPEP3* transcripts per viable cell was relatively low regardless of temperature or media (Figure 1b). Our measurements of cells per ml and transcripts per cell also allowed us to estimate the total yields of *GmPEP3* transcripts per ml of cell culture (Figure 1c), which was significantly impacted by the three-way interaction of growth phase, temperature, and media (*p* = 0.0238). Numerically, the highest transcript levels were observed on 2x YT media at the stationary phase at 30 °C, and for stationary phase cells growth on 2x YT, transcript production was significantly higher at 30 °C than at 37 °C. All stationary phase samples yielded significantly more transcripts/mL of media than log phase samples, indicating that the impacts of higher cell numbers in the stationary phase outweighed the impacts of higher transcripts/cell in the log phase (Figure 1c). Together, these results reveal that growth phase, temperature, and media have interactive effects, and there is a marked tradeoff between total cell production and expression levels per cell, with the log phase promoting higher transcripts per cell and the stationary phase promoting higher cell numbers.

### 2.2. Retention of B. subtilis Endospores and Vegetative Cells with and Without Carboxymethylcellulose on Soybean Seeds

**Figure 2:**
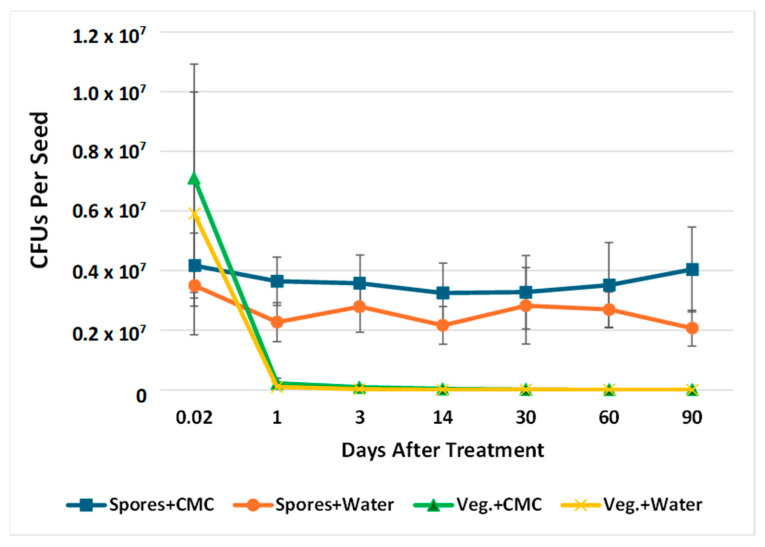
Retention of *B. subtilis* cells on soybean seeds. The number of viable cells reclaimed from seeds treated with spores or vegetative cells (Veg.) with or without CMC between 30 min (0.02 d) and 90 days. Averages for each time point are based on 3 blocks of 3 replicates each (*n* = 9). There was a significant interaction between bacterial cell types and time and between bacterial cell types and CMC (*p* < 0.01), and differences among treatments are discussed in the text and Figure S2.

**Figure 3:**
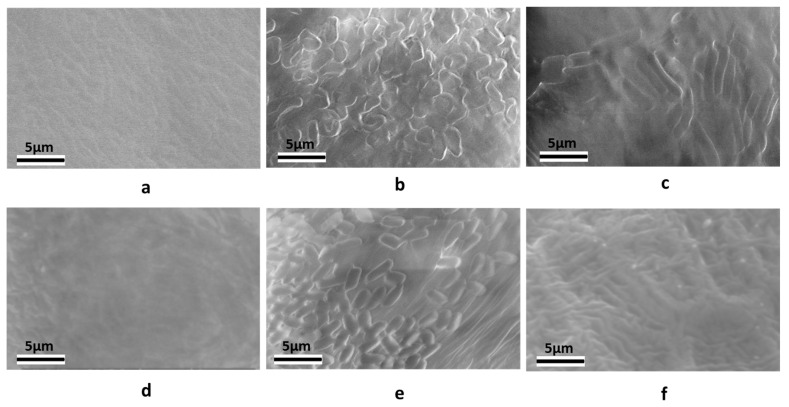
*B. subtilis* spores and vegetative cells on the soybean seed surface on the day of treatment (**a**–**c**) or after long-term storage (**d**–**f**). Scanning electron micrographs (SEM) of seeds treated with (**a**) water, (**b**) spores, or (**c**) vegetative cells 2 h after treatment, or after 5 months of storage (**d**–**f**).

The second goal of this study was to improve the shelf life of *B. subtilis*-seed treatments by comparing the retention of viable spores versus vegetative cells on soybean seeds and assessing the benefits of CMC as an adhesive factor. Soybean seeds were treated with equal concentrations of endospores or vegetative cells of *B. subtilis* expressing *GmPEP3* with or without 0.5% CMC, and then the number of viable bacteria per seed was measured from 30 min to 90 days of storage at 4 °C (Figure 2; see also Figure S2 for information on blocks and separate statistical analyses of each time point). There was a significant interaction between the bacterial cell type and time (*p* < 0.0001). The number of CFUs on seeds treated with vegetative cells dropped precipitously between 30 min and 24 h and remained very low throughout the rest of the experiment (vegetative cells at 30 min compared to all other timepoints: Tukey’s HSD Test, F < 0.0001), whereas the number of CFUs on spore-treated cells remained consistently high from 30 min to 3 months after treatment, and was not significantly impacted by time (all time point comparisons for spore-treated seeds: Tukey’s HSD Test, F ≥ 0.2434). As a result, even though seeds treated with vegetative cells had approximately one-fold higher viable bacterial cells at 30 min than spore-treated seeds (Tukey’s HSD Test, F < 0.0001), spore-treated seeds yielded significantly more CFUs at every subsequent time point (Tukey’s HSD Test, F < 0.0001), with 59-fold higher CFUs at 24 h and >2000-fold higher CFUs at 90 d. There was also a significant interaction between the bacterial cell type and CMC (*p* = 0.0069). Whereas CMC significantly enhanced the number of CFUs on spore-treated seeds (Tukey’s HSD Test, F < 0.0001), it did not help with retention of vegetative cells (Tukey’s HSD Test, F = 0.7880). Seeds treated with CMC in addition to spores on average had at least 16% (at 30 d) and as much as 95% (at 90 d) more viable bacteria than seeds treated with water alone. These results indicate that CMC can increase endospore adhesion to seeds and that endospore seed treatments are stable for at least 3 months. Because the serial dilution plating was performed with selection media, our results also confirm the plasmid carrying the transgene and selection marker was retained during the transition from vegetative growth to endospore production, and over the course of storage. To further confirm differences in the retention of spores versus vegetative cells on seed surfaces, we also used scanning electron microscopy to image both cell types on the surface of treated soybean seeds on the day of treatment or after long-term storage. At 2 h after treatment, bacterial cells could be detected on seeds treated with spores (Figure 3b) or vegetative cells (Figure 3c), but not on water-treated controls (Figure 3a). As expected, vegetative cells were longer and morphologically distinguishable from spores, and each treatment consisted of a uniform cell type (spores or vegetative cells) rather than a mixture. In contrast to the observations at 2 h, after storage, bacterial cells were only detected on seeds treated with spores (Figure 3e), and seeds treated with vegetative cells (Figure 3f) appeared similar to water-treated controls (Figure 3d). These results indicate that the combination of endospores and CMC can dramatically increase the shelf life of *B. subtilis* seed treatments compared to treatments with vegetative cells.

### 2.3. Influence of Seed Treatments with B. subtilis Endospores or Vegetative Cells on Soybean Seedling Growth

**Figure 4:**
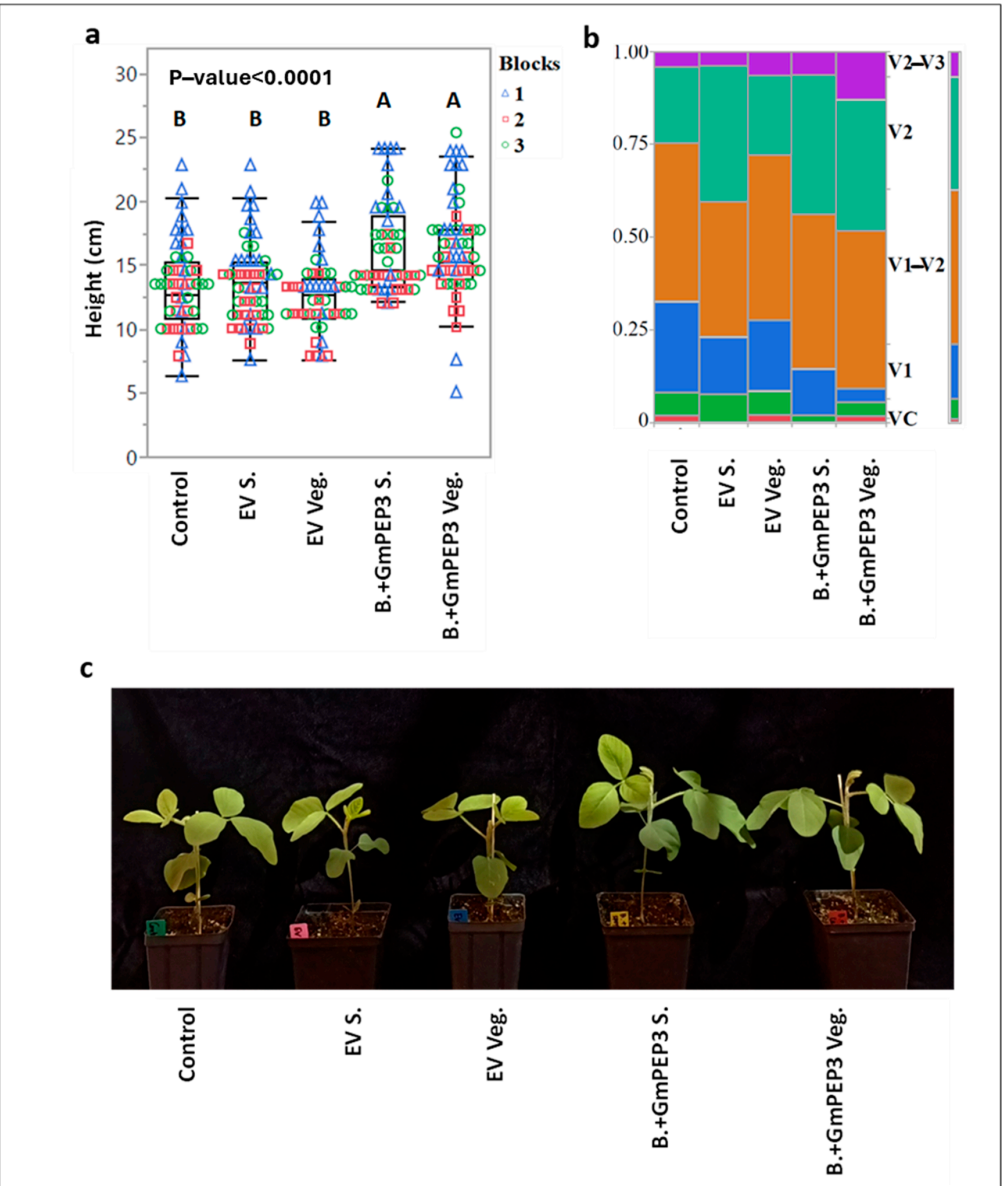
The Effects of Bacterial Seed Treatments on Soybean Seedling Growth. Seeds were treated with spores (S) or vegetative cells (Veg.) of *B. subtilis*, each expressing *GmPEP3* (B + *GmPEP3*) or the empty vector (EV); additional seeds were treated with water as a control. Seeds were then planted, and seedling heights (**a**) and developmental stages (**b**) were measured 17 days after seed germination. Representative plants are shown in (**c**). For seedling heights, the *p* value was generated by a linear mixed model, and treatments labeled with different letters were significantly different according to Tukey–Kramer HSD tests.

Previous work on the benefits of *B. subtilis* seed treatment for soybean was performed with vegetative cells applied during imbibition of seeds immediately prior to planting [14]. In commercial soybean production, however, it is not feasible to imbibe seeds before planting, and seeds must be dried and often stored after treatment. Our results (Figure 2) indicate that endospores, but not vegetative cells can survive drying and storage, suggesting that endospores are more appropriate for seed treatment than vegetative cells. However, to support their use as seed treatments, it is necessary to confirm that endospores have similar bioactivity as vegetative cells. Therefore, we compared the effects of spores and vegetative cells on seedling growth, because prior results indicated that vegetative cell treatments expressing *GmPEP3* increased seedling height at the V1 stage [14]. In the current study, seeds were treated with *B. subtilis* vegetative cells or spores expressing either *GmPEP3* or the empty vector and were planted along with water-treated controls 30 min after treatment to ensure survival of the vegetative cells. The height and developmental stage of each seedling was recorded 17 days after treatment, when nearly all of the water-treated controls had reached the V1 stage or the transition from V1 to V2. There was a significant difference in height among treatments (Mixed Model: *p* < 0.0001), and the treatments that received *B. subtilis* spores or vegetative cells expressing *GmPEP3* were significantly taller than all other treatments (Figure 4a) (Tukey HSD test: *p* ≤ 0.0003 for pairwise comparisons). There was no significant difference between vegetative cells or spores carrying GmPep3 (Tukey HSD test: *p* = 0.9996) or between vegetative cells or spores carrying the empty vector (Tukey HSD test: *p* = 0.5205). *B. subtilis* expressing the empty vector had no significant effect on height compared to water controls (Tukey HSD test: *p* > 0.9 for both spores and vegetative cells), which is consistent with our previous observation that the GmPEP3 peptide but not *B. subtilis* RIK1285 itself influences plant growth [14]. RIK1285 was optimized for ectopic expression rather than plant-growth promoting activity. The effects of ectopic *GmPEP3* expression on seedling height were associated with differences in the plants’ developmental stages. Compared to the empty vector and water-treated controls, by 17 d after planting, more of the seedlings that had received bacteria expressing *GmPEP3* had reached the V2 stage or even the transition between V2 and V3 (Figure 4b,c). Our results indicate that the ectopic *GmPep3* gene is retained in the transition from the vegetative to spore stage, and that treating seeds with genetically modified endospores had similar advantages to plant growth as treatment with viable genetically modified vegetative cells.

## 3. Discussion

Our results reveal that there is a marked trade-off between ectopic expression levels per cell and cell numbers, with the log phase promoting high transcript abundance per viable cell and the stationary phase yielding higher total numbers of viable cells due to longer growth time. In log phase bacteria, the cellular machinery for transcription is highly active to support rapid growth and division, whereas in the stationary phase, after cell numbers have reached their maximum, individual cells have reduced average gene expression levels and metabolic activity to support their long-term survival [32]. Under most combinations of conditions, the stationary phase yielded equal or greater levels of expression per unit of media than the log phase, suggesting that differences in cell numbers had a greater effect on transcript abundance per ml of media than differences in expression levels per cell. The effect of cell numbers may help explain a prior report that production of a heterologous β-agarase enzyme in *B. subtilis* was higher in the stationary phase than in the log phase [17]. We would anticipate, however, that the benefits of the stationary phase for ectopic expression would only be observed with promoters such as *AprE* [28] that are active in this growth phase, as opposed to promoters that are primarily active during rapid growth and down-regulated in the transition to the stationary phase.

Our findings also demonstrate that growth phase, temperature, and media have interactive effects on ectopic gene expression levels in *B. subtilis*. Like harvesting cells in the log phase rather than the stationary phase, growing cells at 30 °C rather than 37 °C favored higher ectopic transcript abundance per cell, and combining these conditions (log phase at 30 °C) had additive benefits on transcription. Previous work on ectopic expression in *Brevibacillus choshinensis* also showed that 30 °C was preferable to 26, 33, and 37 °C [30]. Compared to growth phase and temperature, the choice of media did not have as strong an effect, although it did show significant interactions with the other two conditions. Previously, heterologous enzyme production in bacteria was reported to be greater on 2x YT than LB [18,19], possibly due to increased availability of nutrients from tryptone and yeast extract. In our study, 2x YT promoted higher transcript abundance than LB, but this effect was statistically significant only in the stationary phase grown at 30 °C. Many biological processes in bacteria and other organisms are influenced by the interplay between temperature and nutrient availability [33], which may contribute to the interactive effects observed here.

An alternative to expressing agriculturally useful peptides in *B. subtilis* under laboratory conditions is to inoculate plants with the genetically modified bacteria, enabling the bacteria to deliver the peptides directly to roots [14,16]. Therefore, we also explored approaches to optimize *B. subtilis* seed treatments. The use of endospores dramatically and consistently extended the shelf life of bacterial seed treatments from less than 24 h for vegetative cells to at least 3 months for spore treatments. This agrees with a previous study that reported that *B. cereus* endospores had greater persistence in soil than vegetative cells after soil drenches [34]. In addition, we found that the binding agent CMC significantly increased the number of viable bacteria on seeds treated with spores at all time points, suggesting that CMC facilitates the initial adhesion process. In nature, *B. subtilis* endospores can survive unfavorable conditions for many years, and when exposed to nutrients can germinate within minutes [35]. Therefore, the use of *B. subtilis* spores for seed coating provides a promising method of delivered shelf-stable inoculum to the plant, especially when combined with a polymer that helps adhere bacteria to the seed. Further work is needed, however, to characterize the long-term effects of seed treatments on the bacterial community of the rhizosphere, which can vary depending upon the combination of application method, bacterial and plant species utilized [36,37,38].

Our results also demonstrated that *B. subtilis* endospore treatments had comparable bioactivity as treatments with viable vegetative cells. A previous study on inoculating oat seeds with *Bacillus* spp. reported that vegetative cells were more efficient in promoting seedling growth than endospores, but this difference may have been caused by inequalities in inoculum levels, since vegetative cells had greater adhesion to oat seeds than spores [39]. In contrast, we found that for a *B. subtilis* strain expressing a soybean signaling peptide (*GmPEP3*), spores and vegetative cells benefited seedling growth equally. In addition to establishing the bioactivity of spores, this also illustrates the potential benefits of adding heterologous proteins or peptides to this rhizobacterium’s secretory arsenal.

These findings could facilitate the use of both naturally occurring *B. subtilis* isolates and recombinant strains in crop production. Several commercial biofungicides and biofertilizers have been developed using *B. subtilis* strains GB03 and QST713, and researchers around the world have identified many additional isolates with benefits to plant health [40]. Thus, naturally occurring strains could potentially be developed into new crop protection products through endospore seed treatments. There is also interest in genetically engineering *B. subtilis* or other PGRPs to enhance their efficacy [41]. Because different *B. subtilis* isolates vary in their specificity against plant pathogens as well as their benefits to plant growth and tolerance of abiotic stresses, diverse naturally occurring strains could serve as sources for prospecting peptides or proteins to express in more genetically amenable isolates such as RIK1285. For example, certain strains of *B. subtilis* promote plant growth and stress tolerance by synthesizing and excreting ACC deaminase, an enzyme that reduces reducing ethylene synthesis in plants [13], while others help plants adapt to nutrient limitation and abiotic stresses by secreting phytases that increase phosphate availability or siderophores that facilitate iron uptake [42,43]. In addition, certain strains can reduce plant disease by secreting antimicrobial peptides, chitinases and glucanases that break down fungal cell walls, or lipoproteins that elicit induced systemic resistance (ISR) in the host plant [13,44]. Therefore, increasing the expression levels of individual endogenous genes or pyramiding genes with different bioactivities within a single *B. subtilis* strain could confer a wide range of benefits for crop health. In conclusion, optimizing conditions for ectopic gene expression in *B. subtilis* and its delivery via seed treatments can facilitate its use to promote plant growth, abiotic and biotic stress tolerance.

## 4. Materials and Methods

### 4.1. Biological Materials

This study utilized *B. subtilis* strain RIK1285 that was previously modified to express the mature signaling peptide (*GmPEP3*) encoded by the soybean propeptide gene *GmProPep3* (NM_001248158) under control of the *aprE* promoter (Alnasrawi et al., 2024 [14]). *B. subtilis* RIK1285 originated from Takara Bio, Inc., (San Jose, CA, USA), and excretion of GmPEP3 was directed by the *aprE* signal peptide. *B. subtilis* RIK1285 carrying the pBE-S vector (Takara, San Jose, CA, USA) was used as an empty vector control. The seeds of soybean cultivar *Glycine max* cv. Magellan were used for seed treatment experiments.

### 4.2. Selection of Time Points for Collection of the Log and Stationary Growth Phases of B. subtilis

To identify the time points at which the log phase and stationary phase would occur, bacteria carrying the transgene (*GmPEP3*) or the empty vector were cultured on LB or 2x YT including 10 μg ml^−1^ kanamycin (Sigma-Aldrich, St. Louis, MO, USA), and growth curves were created for each possible combination of media (LB or 2x YT) and temperature (30 °C or 37 °C) using the protocol of [45]. A 96-well plate was treated with 100% ethanol (400 µL per well) for 15 min, and the lid was treated with 10% Triton X for 15 s. to avoid liquid condensation during measurement. Later, the lid and plate were placed under UV for 15 min. 200 µL of a fresh culture grown in LB or 2x YT medium were added to each well with four replicates after adjusting the starting OD600 to 0.05. The absorbance of OD600 readings were measured using the Cytation 3 Multimode plate reader (Biotek, Winooski, VT, USA) every 10 min with continuous shaking for 24 h at 37 °C or 44 h at 30 °C.

### 4.3. Absolute Quantification of Expression

RNA was extracted with TRIZOL™ (Invitrogen, Carlsbad, CA, USA), DNase treated with a TURBO DNA-free™ Kit (Invitrogen, Carlsbad, CA, USA), and reverse-transcribed (1 μg of RNA/sample) using SuperScript TM II Reverse Transcriptase (Invitrogen, Carlsbad, CA, USA;). RT-qPCR was performed using a T100 thermocycler (BIO-RAD, Inc, Hercules, CA, USA) and *GmPEP3* primers: forward 5′-GGGCAAAGGAGGTCAGCATA-3′ and reverse 5′-CTACAGCATCCAGGGTGACG-3′. PCR cycle conditions were as follows: 95 °C for 5 min followed by 30 cycles of 95 °C for 30 s, 55 °C for 30 s and 72 °C for 45 s. Absolute quantification of *GmPEP3* transcripts in experimental samples was calculated by comparison to a standard curve generated using serial dilutions of plasmid DNA ranging from 360 μg/mL to 2900 μg/mL. Plasmid DNA was extracted from recombinant *B. subtilis* using a QIAprep Spin Miniprep Kit (QIAGEN, Germantown, MD, USA). This experiment was repeated twice, and each repetition was treated as a block designated as a random effect in our statistical analysis. Full factorial analysis was performed with a linear mixed model.

### 4.4. Preparation of Endospores

To induce spore formulation, *B. subtilis* expressing *GmPEP3* or the empty vector were cultured in 2xSchaeffer’s glucose (2x SG) medium at 37 °C for three days according to the methods of [46]. One liter of 2x SG medium was prepared with Difco nutrient broth (16.0 g), KCl (2.0 g), and MgSO_4_·7H_2_O (0.5 g). The pH was adjusted to 7.0 and the broth was autoclaved for 20 min at 121 °C. After cooling to 55 °C, 1 M Ca(NO_3_)_2_ (1 mL), 0.1 M MnCl_2_·H_2_O (1 mL), 1 mM FeSO_4_ (1 mL) and 50% (*w*/*v*) filter-sterilized glucose (2 mL) solutions were added to the medium [47]. To generate a starter culture, a fresh colony of *B. subtilis* expressing GmPEP3 (B. + GmPEP3) was inoculated in 10 mL of LB medium with kanamycin (10 μg ml^−1^) and incubated at 37 °C with shaking at 200 rpm overnight. Then, to induce spore formulation, 1 mL of the overnight culture in LB was added to 200 mL of 2x SG medium and left to grow by shaking at 200 rpm at 37 °C for three days [46]. The cells were centrifuged at 8000 for 15 min at 4 °C. and stored at −20 °C to be used for seed coating. The cells were then centrifuged at 8000 for 15 min at 4 °C. and stored at −20 °C to be used for seed coating. To confirm the presence of spores and the absence of vegetative cells, bacterial smears were fixed with low heat, stained with 1% malachite green for 10 min, rinsed with water, counterstained using 1% aqueous safranin for 30 s,. and examined using an Olympus BX51 light microscope with a 100× oil immersion objective lens (Olympus Corporation, Hachioji, Tokyo), similar to methods described by [48]. This method stains endospores green and vegetative cells pink, and our observations confirmed that 2x SG yielded homogeneous endospore cultures, while LB yielded homogeneous vegetative cell cultures.

### 4.5. Bacterial Seed Treatments

Prior to treatment, soybean seeds were surface-sterilized using 10% sodium hypochlorite (i.e., bleach) for 1 min followed by 70% ethanol for 3 min and then washed 3–5 times with autoclaved water. Suspensions of vegetative cells were prepared from overnight culture in LB supplemented with kanamycin (10 μg ml^−1^). Vegetative cells with OD600 ~0.7 were centrifuged and prepared by removing the LB and adding the corresponding volume of distilled water or 0.5% CMC. To coat seeds with a spore suspension, water or 0.5% CMC was added to spore cells to reach OD600 ~0.7 [49]. Seeds were mixed with vegetative cells or spores at 1:5 (*v*/*w*) ratio, then air dried for 30 min at room temperature. This experiment was repeated three times, and each repetition was treated as a block designated as a random effect in our statistical analysis. Full factorial analysis was performed with a linear mixed model.

### 4.6. Measurement of Retention of Viable Bacteria on Treated Seeds

At 30 min after seeds were treated with either vegetative cells or spores of *B. subtilis* expressing *GmPEP3*, three samples (each consisting of a pool of five seeds) from every treatment group were placed in normal saline solution to a final volume 5 mL. Serial dilutions were made and plated to count to CFUs for every treatment. The remaining seeds were collected in a 50 mL conical tube and stored at 4 °C to be checked at 24 h, 72 h, 14 d, 30 d, 60 d, and 90 d after treatment.

### 4.7. Measurement of Seedling Growth After Bacterial Seed Treatments

Seeds were coated with vegetative cells or spores of *B. subtilis* expressing either *GmPEP3* (B. + GmPEP3) or the empty vector (EV) as described above. After air-drying, the coated seeds were wrapped in moist germination paper and then transplanted into soil (LC1 Sunshine Mix, Sungro Horticulture, Agawam, MA, USA) after germination. The heights of the plants were measured manually at 17 days after seed germination, and the developmental stages of the plants were also recorded. We defined the vegetative stages according to the soybean growth stage descriptions by [50]. Cotyledons emerge from the soil surface is defined as VE stage; unifoliate leaves are fully expanded when the plants are VC stage; The first trifoliate are fully emerged and opened while the plants are V1 stage; plants having three nodes with two trifoliate fully opened are V2 stage; plants having four nodes with three trifoliate fully unfolded are V3 stage (Figure S3). This experiment was repeated three times, and each repetition was treated as a block designated as a random effect in our statistical analysis. One way analysis was performed with a linear mixed model.

### 4.8. Micrographs of B. subtilis Spores and Vegetative Cells on the Soybean Seed Surface

Seeds were treated with spores or vegetative cells expressing GmPep3 (no CMC) as described in Section 4.5. Additional controls were treated with water only. The seeds were either imaged after air-drying (2 h after treatment), or after long-term storage (5 months at 4 °C). The seed surfaces were observed using an Environmental Scanning Electron Microscope (XL-30 ESEM, Philips Semiconductors, Eindhoven, The Netherlands) in order to confirm the presence of *B. subtilis* spores and vegetative cells.

### 4.9. Statistical Analysis

All the data were analyzed using JMP Pro 17.0 software (SAS Institute, Cary, NC, USA). Since each experiment was performed more than once, each run was treated as a block and blocks were treated as a random effect factor. Linear mixed models were used to detect significant main effects of the independent variables, as well as potential interactions among all independent variables. When interactions were detected, Tukey’s Honestly Significant Difference (HSD) mean separation tests were used to determine which treatments differed from one another. An α value of 0.05 was used for all tests.

## Data Availability

The original contributions presented in this study are included in the article/supplementary material. Further inquiries can be directed to the corresponding author.

## Supplementary Materials

The following supporting information can be downloaded at: https://www.mdpi.com/article/10.3390/plants14203184/s1: Figure S1: Growth Curve for *B. subtilis*, Figure S2: Retention of *B. subtilis* cells on soybean seeds, Figure S3: Soybean development stage.

## Abbreviations

The following abbreviations are used in this manuscript:

2x YT	Yeast Extract Tryptone media
LB	Luria–Bertani (LB) media
CFU	Colony forming units
CMC	Carboxymethylcellulose
