# Supplementary material for: Optimizing Conditions for Bacillus subtilis Ectopic Gene Expression and Delivery via Seed Treatment
Source: Plants (Basel). 2025 Oct 16;14(20):3184. doi: 10.3390/plants14203184 (PMC12567175; doi:10.3390/plants14203184)
Supplement: Supplementary file 1 [file plants-14-03184-s001.zip › plants-3879712-supplementary.pdf]

SUPPLEMENTARY MATERIALS

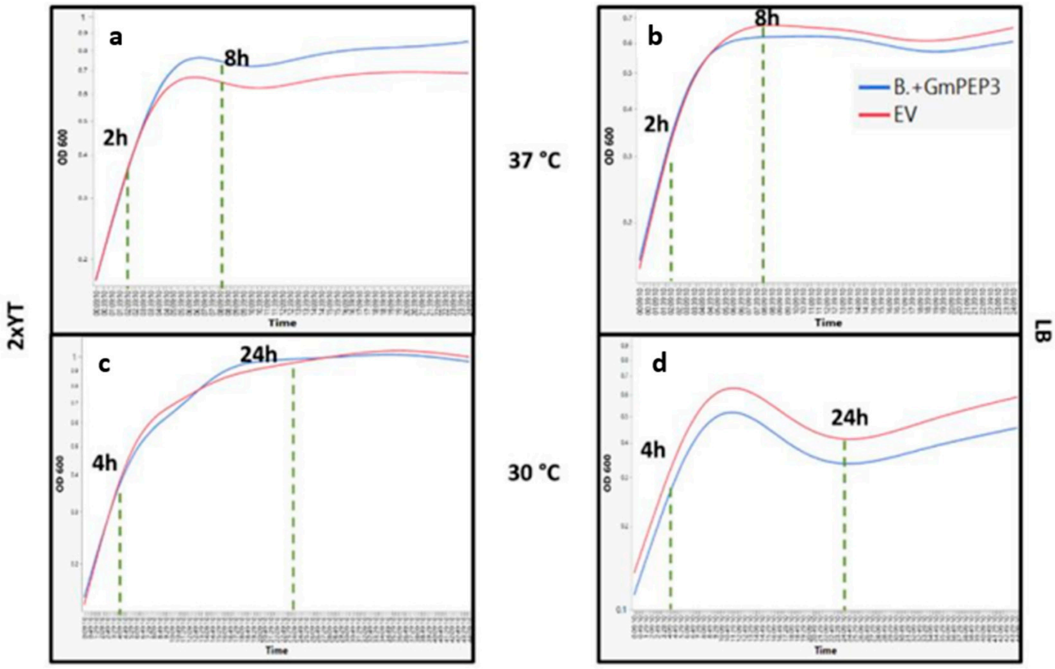

Figure S1. Growth curve for *B. subtilis*. Growth curves were constructed to determine the time points at which the log phase (LF) and the stationary phase (SF) occur for *B. subtilis* when cultured at 37 °C on 2x YT (a) or LB (b), or at 30 °C on 2x YT (c) or LB (d). Growth curves were measured for *B. subtilis* expressing *GmPEP3* (B.+GmPEP3, blue lines) and for *B. subtilis* carrying the empty vector (EV, red lines) to confirm that the GmPEP3 transgene did not impact bacterial growth. (as a control) in cultured in LB or 2x YT medium with kanamycin (10 µg ml^{-1}) at 37 °C or 30 °C. OD600 readings were measured every 10 min for 24 hrs at 37 °C or 44 hrs at 30 °C. n=4.

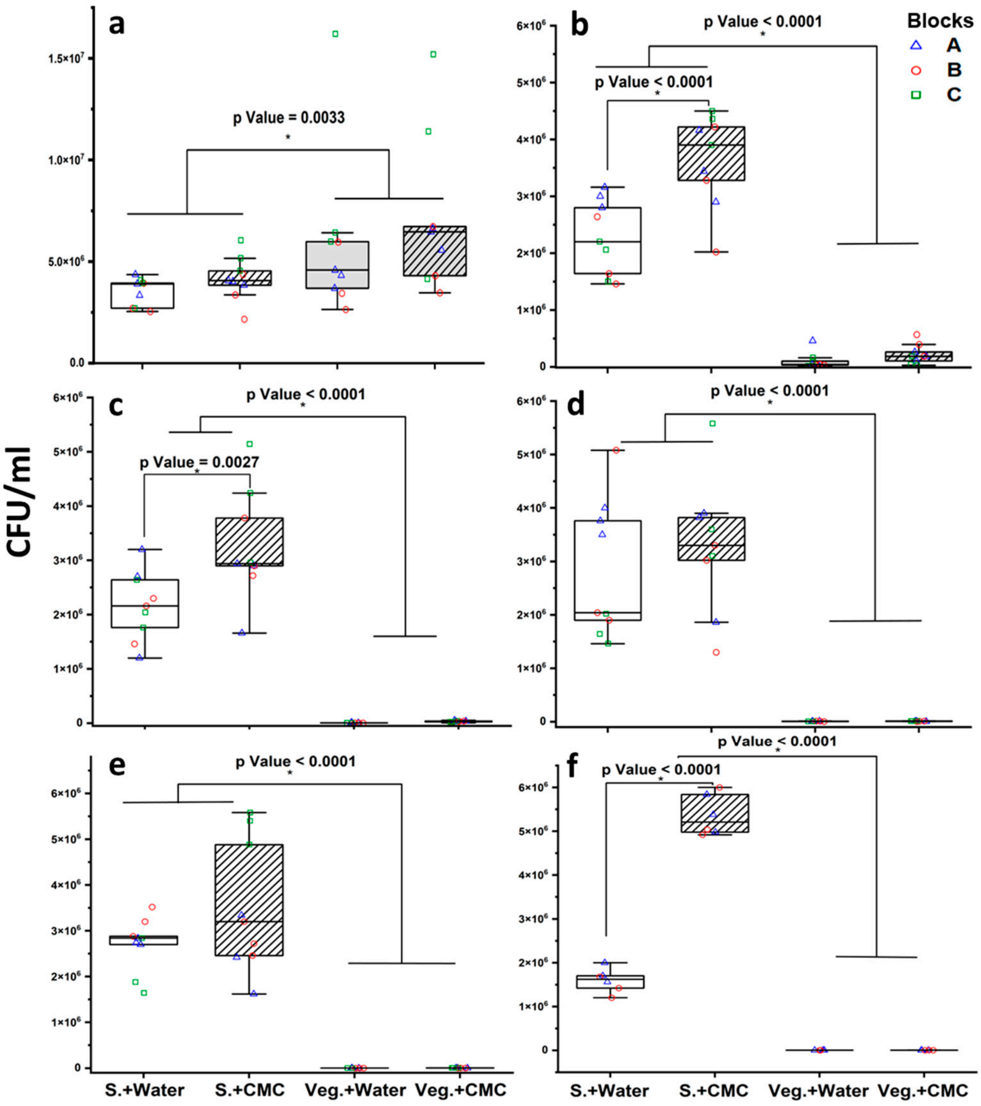

Figure S2. Retention of *B. subtilis* cells on soybean seeds. The number of viable cells reclaimed from seeds treated with spores (S) or vegetative cells (Veg.) with (cross-hatched bars) or without (open bars) CMC was tested at 30 min (a), 24 hrs (b), 2 weeks (c), 30 d (d), 60 d (e), and 90 d (f) after seed treatment using serial dilutions. The experiment included 3 blocks (3 biological replicates each) replicated in time (N = 9). Each time point was analyzed separately and the P values for main effects with linear mixed models. When time points were analyzed separately, no significant interactions among the main effects were observed (Prob > F = > 0.05).

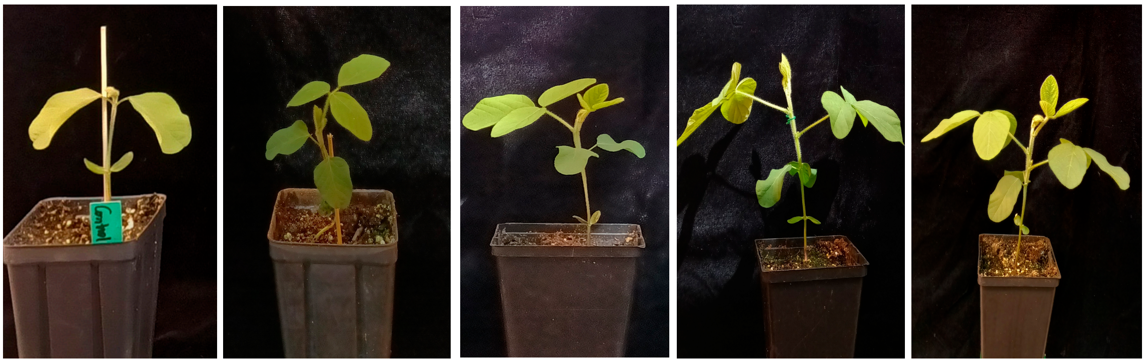

a b c d e

Figure S3. The Soybean Development Stages. a) VC stage. The unifoliate leaves are fully expanded; b) V1 stage. The first trifoliates are fully emerged and opened; c) V1-V2 stage between V1 and V2 stage; d) V2 stage. Plants having three nodes with two trifoliate are fully opened; e) V3 stage. Plants having four nodes with three trifoliate are fully unfolded.
